# NFAT2 overexpression suppresses the malignancy of hepatocellular carcinoma through inducing Egr2 expression

**DOI:** 10.1186/s12885-020-07474-0

**Published:** 2020-10-06

**Authors:** Jian Wang, Yamin Zhang, Lei Liu, Zilin Cui, Rui Shi, Jiancun Hou, Zirong Liu, Long Yang, Lianjiang Wang, Yang Li

**Affiliations:** 1 Hepatobiliary Surgery Department, Tianjin First Center Hospital, Tianjin Clinical Research Center for Organ Transplantation, Key Laboratory for Critical Care Medicine of the Ministry of Health, No. 24 Fukang Road, Nankai District, Tianjin, 300192 PR China; 2 Department of Transplantation Center, Tianjin First Center Hospital, Tianjin Clinical Research Center for Organ Transplantation, Key Laboratory for Critical Care Medicine of the Ministry of Health, Tianjin, 300192 PR China

**Keywords:** NFAT2, HepG2 cells, Hepatocellular carcinoma, Cancer malignancy

## Abstract

**Background:**

Nuclear factor of activated T cells 2 (NFAT2) has been reported to regulate the development and malignancy of few tumors. In this study, we aimed to explore the effect of NFAT2 expression on cell fate of HepG2 cell and its potential mechanisms.

**Methods:**

Firstly, the pcDNA3.1-NFAT2 plasmid was transfected into HepG2 cells to construct NFAT2 overexpressed HepG2 cells. Then, the chemical count kit-8 cell viability assay, Annexin V-FITC apoptosis detection, EdU labeling proliferation detection, transwell and wound healing experiments were performed. The expression of Egr2 and FasL, and the phosphorylation of AKT and ERK, after ionomycin and PMA co-stimulation, was detected, while the Ca^2+^ mobilization stimulated by K^+^ solution was determined. At last, the mRNA and protein expression of NFAT2, Egr2, FasL, COX-2 and c-myc in carcinoma and adjacent tissues was investigated.

**Results:**

The NFAT2 overexpression suppressed the cell viability, invasion and migration capabilities, and promoted apoptosis of HepG2 cells. NFAT2 overexpression induced the expression of Egr2 and FasL and suppressed the phosphorylation of AKT and ERK. The sensitivity and Ca^2+^ mobilization of HepG2 cells was also inhibited by NFAT2 overexpression. Compared with adjacent tissues, the carcinoma tissues expressed less NFAT2, Egr2, FasL and more COX-2 and c-myc.

**Conclusion:**

The current study firstly suggested that NFAT2 suppressed the aggression and malignancy of HepG2 cells through inducing the expression of Egr2. The absence of NFAT2 and Egr2 in carcinoma tissues reminded us that NFAT2 may be a promising therapeutic target for hepatocellular carcinoma treatment.

## Background

Hepatocellular carcinoma (HCC) has become the leading cause of deaths that are caused by all cancers worldwide [1] and the incidence and mortality of HCC are increasing at a faster pace than other cancers, which trend will continue through at least 2030 in United States [2]. The factors, such as hepatitis C virus infection, obesity, type II diabetes and alcohol drinking, all contribute to the initiation of HCC [2]. Although some improvements in prevention, detection and treatment of HCC have developed in the past decades, only 1/5 patients could survive to 5 years after diagnose [3]. Therefore, research on hepatocellular transformations in initiation and development of HCC and identifying effective biomarkers for novel therapeutic option are still emergency challenges.

Nuclear factor of activated T cells 2 (NFAT2, also known as NFATc and NFATc1) is an important member of NFAT family which plays a vital role in T cell activation and differentiation [4]. Generally, NFAT2 locates in cytoplasm with an hyperphosphorylation in quiescent cells, while it can be activated through dephosphorylating by intracellular Ca^2+^ increase activated calcineurin [5]. Once NFAT2 is activated, it exposes nuclear localization sequences, promoting its cytoplasm-to-nuclear translocation and then NFAT2 forms heterodimers with other transcription factors to exert its transcriptional functions [6]. NFAT2, as an important transcriptional promoter, regulates expression of TNF-α, myc proto-oncogene protein (c-myc), cyclooxygenase-2 (COX-2), Fas ligand (FasL) and also generates crosstalks with ERK/MAPK pathway and AKT/GSK3β signaling, which achieves its control of the cell fate [7–12]. Therefore, besides the regulation of immune cells, NFAT2 also exerts modulatory effect of proliferation, invasion, metastasis and malignancy in breast cancer, lung cancer, melanoma and leukemia [13–15]. Furthermore, NFAT2 participates in the early growth response factor 2 (Egr2)-regulated anergic phenotype which impairs the migration and invasion of leukaemia cells [16]. Due to the complex functions of NFAT2, the role of NFAT2 in HCC has not been clearly clarified and the existed research data displays completely opposite effect of NFAT2 in HCC [17, 18] Therefore, more research on the effect of NFAT2 in HCC is necessary for profoundly understanding the potential role of NFAT2 in HCC therapy.

In this study, we constructed NFAT2 overexpressed cells (HepG2/NFAT2) based on HepG2 and investigated the effect of NFAT2 overexpression on HepG2 cells’ viability, apoptosis, proliferation, invasion and migration. The expression levels of NFAT2, Egr2, FasL and the phosphorylation of AKT and ERK were detected after ionomycin stimulation. The expressions of NFAT2, Egr2, FasL, COX-2 and c-myc were examined in tissues from HCC patients. When HepG2/NFAT2 and NC cells stimulated by high K^+^ solution, the Ca^2+^ mobilization was monitored by Live Cell Imaging System to evaluate the sensitivity of cells to K^+^ irritation. Our study demonstrates that NFAT2 is a promising HCC inhibitor for its regulation on sensitivity and aggression of HepG2 cells to cytokines’ stimulation, through modulating the expression of anergy-associated and antitumor transcription factor, Egr2.

## Methods

### Materials

The expression plasmids pcDNA3.1-NFAT2 and its empty vector pcDNA3.1 were purchased from promega (WI, USA). The Lipofectamine 2000 was obtained from Invitrogen (Thermofisher Scientific., USA). Pluronic F-127 was bought from Genecopoeia (MA, USA). Fluo-4/AM, anhydrous dimethyl sulfoxide (DMSO) and cell counting kit-8 (CCK-8) were acquired from Dojindo Molecular Technologies Inc. (Tokyo, Japan). Antibodies against NFAT2 (RRID: AB_2152507), Egr2 (RRID:AB_1139730), FasL (RRID:AB_302235), ERK (RRID:AB_11157324) and β-actin (RRID:AB_764434) were obtained from Abcam (Cambridge, UK). Antibodies against AKT (RRID:AB_329827), p-AKT(Try326) (RRID:AB_1264114), p-ERK(S189) (RRID:AB_490903), c-myc (RRID:AB_2151827) and Cox-2 (RRID:AB_2084968) were bought from Cell Signaling Technology (MA, USA). The HRP-conjugated secondary antibody was acquired from Bioss, Inc. (Beijing, China). Annexin V-FITC apoptosis detection kit was obtained from BD Biosciences (NJ, USA). The Click-iT Plus 5-ethynyl-2′-deoxyuridine (EdU) Flow Cytometry Assay kit was bought from Invitrogen. All chemical reagents used in this study were of analytical grade and all reagents for cell culture were purchased from Gibco BRL Life Technologies (MD, USA).

### Cell culture and tissue samples

Human liver hepatocellular carcinoma (HepG2) cells (RRID:CVCL_0027) which were bought from American Type Culture Collection (ATCC, Rockville, MD, USA) were cultured in Dulbecco’s modified Eagle’s medium (DMEM) including 10%(V/V) fetal bovine serum (FBS), 0.1 mg/mL streptomycin and 100 U/mL penicillin in a 37 °C humidified incubator with 5% CO_2_. Cells were fed every day and were sub-cultured once they reached 90% confluency.

Hepatic carcinoma samples, including carcinoma samples (CS) and adjacent nontumor samples (AS) were obtained from 20 patients who were diagnosed as HCC and received hepatectomy during 2018 at the Tianjin First Central Hospital. The age of those patients ranged from 20 to 78 years. The tissue samples were stored at liquid nitrogen for PCR and western blot assay.

### Transient transfection assay

HepG2 cells were cultured in 6-well plate with complete medium for 24 h. Then, the cells were respectively transfected with 70 nmol/L plasmids pcDNA3.1-NFAT2 and the control vector, pcDNA3.1 in fresh serum free medium for 24 h. Lipofectamine 2000 was used in the transfection according to the manufacturer’s instructions. All the cells stably transfected with pcDNA3.1-NFAT2 were confirmed by western blot and RT-qPCR analysis and designated as HepG2/NFAT2. The cells stably transfected with pcDNA3.1 vector were regarded as negative control (NC). The HepG2/NFAT2 and NC cells were sub-cultured and applied for the following analysis.

### Cell counting kit-8 (CCK-8) assay

The CCK-8 assay was performed to detect the viability of HepG2/NFAT2 and NC cells. The cells, in brief, were seeded into 96-well plate and were cultured in 200 μL complete medium for 12, 24, 48 and 72 h, respectively. Then, CCK-8 reagents were respectively added to the medium of corresponding wells and the cells were incubated at 37 °C for another 2 h. Subsequently, the absorbance of each well was monitored at optical density (OD) 450 nm by Microplate Reader (Bio-Tek Instruments, VT, USA).

### Annexin V-FITC staining and EdU labeling assay

The cells were seeded in a 6-well plate at a density of 2 × 10^5^ cells/well, with the complete medium overnight. The cells were collected, washed by ice-cold PBS for three times and then resuspended by 1 × binding buffer. 100 μL cell suspension was stained by 1/1000 (v/v) Annexin V-FITC for 20 min at room temperature, in the dark. Next, the cells were washed thoroughly by PBS and stained with 2 μg/mL propidine iodide (PI) in deliquated binding buffer. The apoptosis evaluation was performed by flow cytometer (Beckman Coulter, CA, USA) and analyzed by FlowJo software (RRID:SCR_008520) (Tree Star, OR, USA).

To assess the cell proliferation, the EdU (5-ethynyl-2′-deoxyuridine) labeling assay was performed by incubating NC and HepG2/NFAT2 cells with serum free medium containing 10 μmol/L EdU for 2 h. Then, the cells were washed by PBS and fixed for 15 min at room temperature. The fixed cells were washed and re-suspended in permeabilization buffer for 10 min in dark place. The cell suspension was incubated in Click-iT plus reaction cocktail for 30 min at room temperature in the dark. The stained cells were washed and analyzed by flow cytometer.

### Transwell assay

To evaluate the invasion efficiency, the transwell assays were conducted in 24-well transwell inserts (8.0 μm pores, BD Biosciences) according to the recommendations by the manufacturer. Briefly, cells (1 × 10^5^ cells/chamber) were plated in the upper chamber, with serum free medium while medium containing 10% FBS, using as chemoattractant, was placed in the lower chamber. After 24 h incubation, migrated cells in the bottom surface of the upper chamber membranes were fixed by methanol for 5 min and then stained with 0.1% crystal violet in 20% ethanol for 15 min. The random visual fields were imaged by a light microscope. The values of invasion were expressed with the mean absorbance of OD 570 nm per assay.

### Wound healing assay

The wound healing assays were applied for assessing the migration capacity of cells. Cells were seeded into 6-well plates with complete medium, at a density of 5 × 10^5^ cells/well and then cultured for 24 h. The linear wounds were scratched by a pipette tip on the completely confluent monolayer cells, timed 0 h and then cells were cultured in serum free medium for 24 h, the last moment timed 24 h. The images at 0 h and 24 h were captured by an inverted microscope and the migration % = (the distance of two sides at 0 h (D0) – the distance of 24 h (D24))/D0.

### Fluo-4-Ca^2+^ complex analysis

HepG2 cells were cultured in a 6-well plate for 24 h and then incubated by hank’s balanced salt solution (HBSS, without Ca^2+^ and Mg^2+^) containing 1 μmol/L fluo-4/AM and 0.5‰ pluronic F-127 for 20 min at incubator. After fluo-4/AM loaded, the cells were washed by HBSS for three times and then equilibrated in HBSS for 30 min in the dark. The fluorescence intensity of fluo-4-Ca^2+^ complex was detected by a Live Cell Imaging System (AF7000, Leica, Germany) with Ex 488 nm and Em 516 nm, and 20 mmol/L KCl was added into wells at almost 15 s. The fluorescence curve was calculated by ∆F/F_0_ = (the mean fluorescence intensity of cells – the background intensity)/the background intensity.

### qRT-PCR

Total RNAs in cells and tissues were extracted by TRIzol™ Plus RNA purification kit (Invitrogen) and first-strand cDNA was synthesized by the ImProm-II reverse transcription system (Promega). Real-time PCR assays were conducted by the SYBR Green Mastermix (Applied Biosystems) and all procedures were performed according to the manufacturer’s instructions. Specific primers used for genes were as follows: NFAT2 forward, 5′-GCTATGCATCCTCCAACGTC-3′; and reverse, 5′-AGTTFFACTCGTAGGAGGAG-3′; EGR2 forward, 5′-TCAGCATCTCCCAACCTAT-3′; and reverse, 5′-ACAACAAACACTACCACCCT-3′; FASL forward, 5′-GTTCTGGTTGCCTTGGTAG-3′; and reverse, 5′-CATCTGGCTGGTAGACTCT-3′; COX-2 forward, 5′-GAAAGCCCTCTACCATGACATC-3′; and reverse, 5′-CACCCTTTCACATTATTGCAGA-3′; c-myc forward, 5′-GCCACGTCTCCACACATCAG-3′; and reverse, 5′-TCTTGGCAGCAGGATAGTCCTT-3′; β-actin forward, 5′-CTGGGACGACATGGAGAAAA-3′; and reverse, 5′-AAGGAAGGCTGGAAGAGTGC-3′. All data were analyzed by the ABI7300 system SDS software (Applied Biosystems) and the mRNA relative expression was calculated by the 2^-∆∆Ct^ method. The β-actin expression was regarded as control, so the expression value for each gene was normalized to β-actin expression in each sample.

### Cell treatment

The cells were seeded in a 6-well plate at a density of 2 × 10^5^ cells/well, with the complete medium overnight. 1 × 10^− 3^ mol/L ionomycin and 1 mg/mL 12-myristate 13-acetate (PMA) were applied to pretreat cells for 8 h. The cells were then washed and subjected to western blot analysis.

### Western blot

Total proteins in cells were extracted by ice cold RIPA lysis buffer added PMSF for 30 min. While the process of hepatic protein extraction was performed by firstly pulverizing hepatic tissues in liquid nitrogen and lysing the fragments in ice cold RIPA buffer for 30 min. The protein extractions were centrifugated at 15000 rpm at 4 °C for 10 min and the supernatants were quantified by bicinchoninic acid protein assay kit (Thermo Fisher Scientific). The equivalent protein samples were subjected to 10% SDS-PAGE followed by western blot analysis. The PVDF membranes were blocked by 5% skim milk in TBST for 1 h at room temperature, and next were incubated overnight at 4 °C by the primary antibodies diluted in TBST: anti- NFAT2 (1:1000), Egr2 (1:1000), FasL (1:2000), AKT (1:1000), p-AKT (1:1000), ERK (1:1000), p-ERK (1:1000), COX-2 (1:1000), c-myc (1:2000), and β-Actin (1:1000). After primary antibodies incubation, the PVDF membranes were incubated with HRP-conjugated secondary antibody for 1 h at room temperature. The proteins were stained with Immobilon™ Western Chemiluminescent HRP Substrate detection reagent (Millipore, MA, USA) and imaged using Image Lab™ software (Bio-Rad, VA, USA). To quantify the relative intensity of proteins, the ratios of target proteins to β-actin signal were calculated by ImageJ software. The full-length bots/gels are presented in the Supplementary Fig. 2.

### Immunohistochemistry (IHC)

Paraffin sections were deparaffinized in xylene and then successively rehydrated by graded alcohol. Antigen retrieval was conducted through boiling the section samples in EDTA (pH 6.0) in a microwave oven at 95 °C for 10 min. The section samples were incubated in 3% hydrogen peroxide for 30 min at room temperature and then blocked by 20% goat serum for 40 min. Section samples were incubated with anti- NFAT2 antibody in 20% goat serum (1:100) overnight at 4 °C. All sections were incubated by HRP-conjugated secondary antibody for 60 min at room temperature. At last, the protein was stained by diaminobenzidine and the sections were counterstained with hematoxylin.

### Statistical analysis

Statistical analysis was performed using SPSS 24.0 (RRID:SCR_002865) (SPSS Inc., Chicago, USA) and all values are presented as the mean ± standard deviation (SD). A two-tailed, student’s *t*-test was used to statistical comparison between two groups. For data that didn’t distribute normally, a paired two-tailed Wilcoxon matched-pairs signed-rank test or a two-tailed unpaired Mann-Whitney test were applied to calculate its significance. The *p* values less than 0.05 was regarded as statistically significant.

## Results

### pcDNA3.1-NFAT2 upregulated the expression of NFAT2 in HepG2 cells

**Fig. 1:**
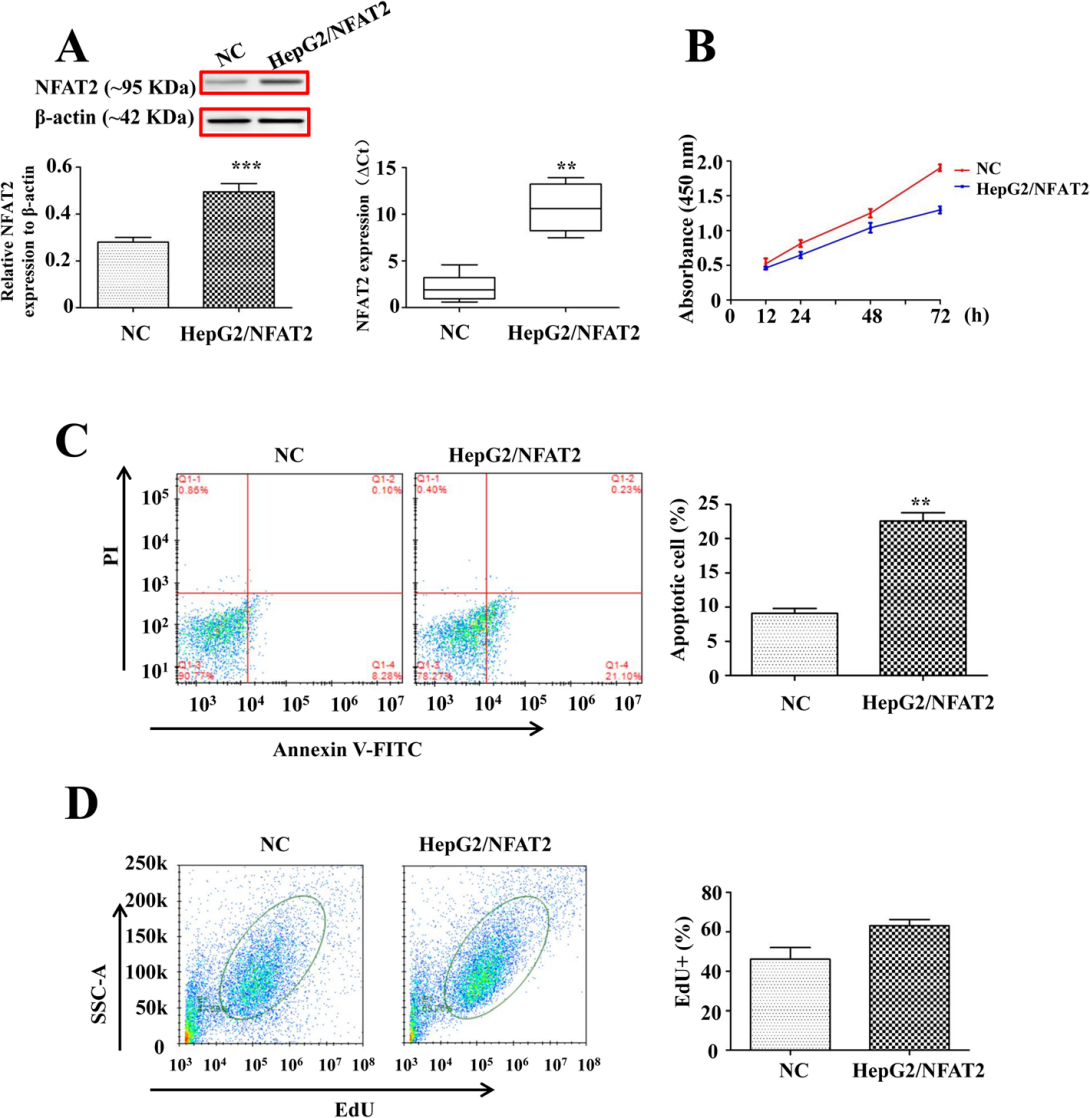
NFAT2 overexpression suppressed cell viability and induced cell apoptosis in HepG2 cells. **a** Western blot and qRT-PCR analysis of protein and gene expression of NFAT2 in HepG2 cells transfected with pcDNA3.1-NFAT2 and pcDNA3.1 plasmids (*n* = 3). **b** The cell viability of NC and HepG2/NFAT2 cells was represented by absorbance values (λ = 450 nm), detected by CCK-8 kit (*n* = 6). **c** The cell apoptosis assay of NC and HepG2/NFAT2 cells by Annexin V-FITC and PI staining (*n* = 6). **d** The cell proliferation assay of NC and HepG2/NFAT2 cells by EdU labeling (*n* = 6). Each bar represents the mean ± S.D.; ^*^p < 0.05, ^**^p < 0.01, ^***^p < 0.001 compared to the NC samples. Full-length blots are presented in Supplementary Figures

To construct NFAT2 highly expressed cells, the pcDNA3.1-NFAT2 plasmid and its control vector were transfected into HepG2 cells. Then, the relative expression of NFAT2 in NC and HepG2/NFAT2 cells was evaluated by qRT-PCR and western blot. The results revealed that NFAT2 protein and mRNA expression was effectively upregulated in HepG2/NFAT2 cells when compared with NC (Fig. 1a). Therefore, the HepG2/NFAT2 and NC cells were applied to investigate the influence of NFAT2 overexpression on tumor cells behavior.

### NFAT2 overexpression suppressed viability of HepG2 cells

In this study, the CCK-8 kit was used to determine the cell viability of HepG2/NFAT2 and NC cells, and the more absorbance of CCK-8, the higher cell viability of HepG2. The absorbance of CCK-8 in HepG2/NFAT2 cells was less than that in NC cells from 12 h to 72 h and compared to NC cells, this absorbance of HepG2/NFAT2 cells, was remarkably decreased at 72 h (Fig. 1b), which indicated that NFAT2 overexpression in HepG2 cells could suppress the cell viability.

### NFAT2 overexpression induced apoptosis of HepG2 cells

The early apoptotic cells were stained only by Annexin V and located in the Q1–4 area in Fig. 1c, while the late apoptotic cells were stained by Annexin and PI, and located in the Q1–2 area. The early and late apoptotic cells were all included in cell apoptosis evaluation. The percentage of apoptotic HepG2/NFAT2 cells was greatly higher than that of apoptotic NC cells (Fig. 1c), which indicated that NFAT2 overexpression promoted apoptosis of HepG2 cells.

### NFAT2 overexpression didn’t regulate the proliferation of HepG2 cells

To investigate the influence of NFAT2 overexpression on HepG2 cell cycle progression, the EdU incorporation assay was adopted. The cells with positive EdU labeling were proliferating actively. The results in Fig. 1d revealed that the percentages of EdU-positive cells in HepG2/NFAT2 and NC cells were not significantly different. Therefore, NFAT2 overexpression in this study, didn’t exert obvious effect on proliferation of HepG2 cells.

### NFAT2 overexpression inhibited HepG2 cells’ invasion and migration

**Fig. 2:**
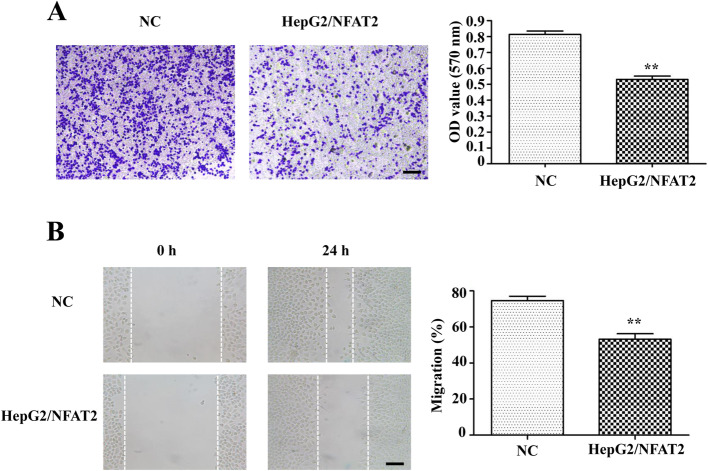
NFAT2 overexpression inhibited the invasion and migration capability of HepG2 cells. **a** The images and quantitative analysis of invaded NC and HepG2/NFAT2 cells by transwell analysis (*n* = 6). (Scale bars = 200 μm; Magnification, 10×). **b** The images and quantitative analysis of migrated NC and HepG2/NFAT2 cells by wound healing analysis (*n* = 6). (Scale bars = 200 μm; Magnification, 10×). Each bar represents the mean ± S.D.; ^*^p < 0.05, ^**^p < 0.01, ^***^p < 0.001 compared to the NC samples

To assess the influence of NFAT2 overexpression on invasion capability of HepG2 cells, the transwell assay was conducted. The HepG2/NFAT2 cells exerted weak invasion capability with less absorbance at 570 nm (Fig. 2a), compared with NC cells. The results in Fig. 2b, showed that the migration (%) was decreased in NFAT2 overexpressed HepG2 cells. Thus, NFAT2 overexpression in HepG2 cells exerted suppression on the cell invasion and migration potential.

### NFAT2 overexpression promoted the expression of Egr2, FasL and suppressed the phosphorylation of AKT and ERK

**Fig. 3:**
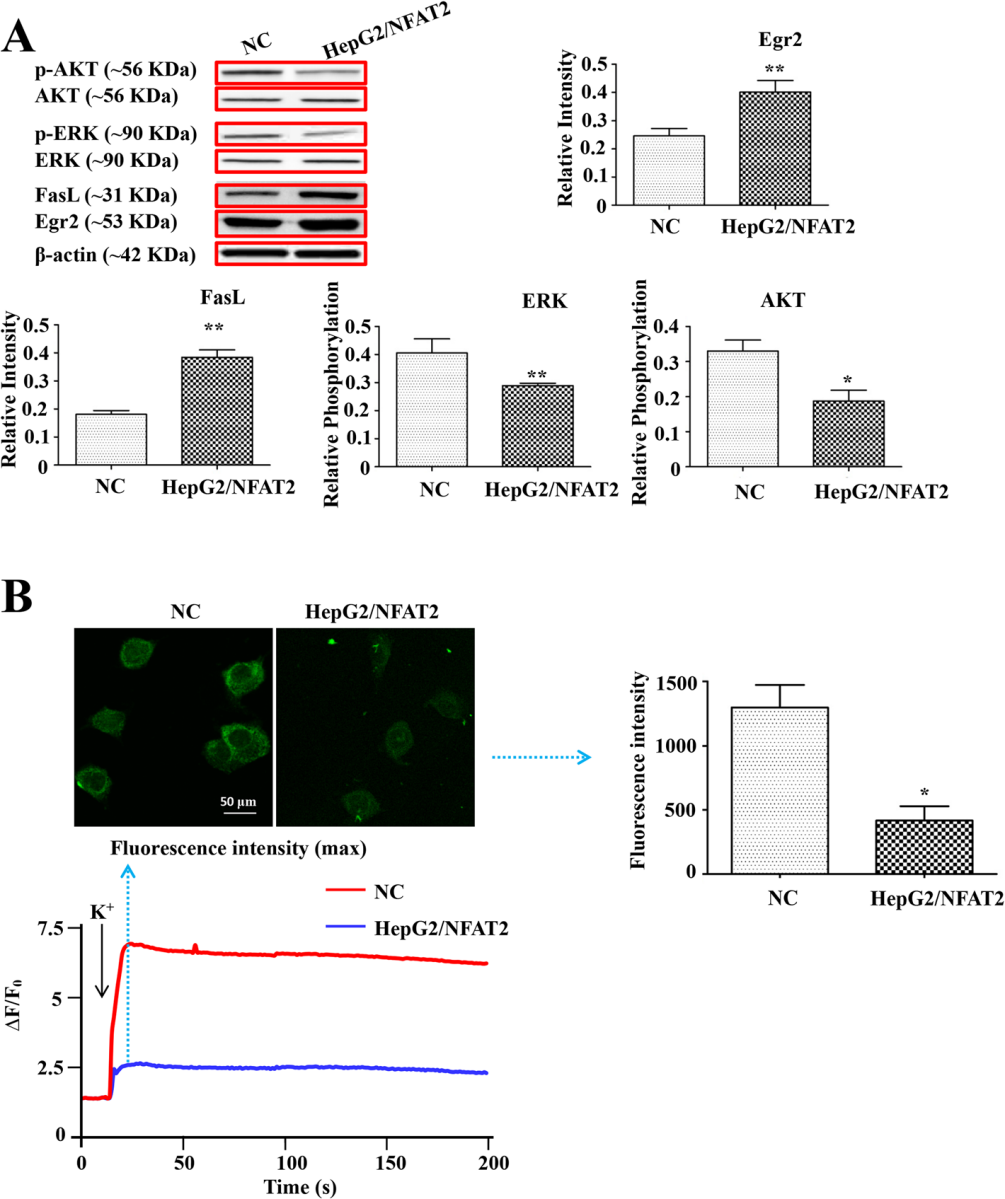
NFAT2 overexpression induced the expression of Egr2, FasL and suppressed the phosphorylation of AKT and ERK, and Ca^2+^ mobilization in HepG2 cells. **a** The protein expression of Egr2, FasL, AKT, p-AKT, ERK and p-ERK in NC and HepG2/NFAT2 cells, was detected by western blots (*n* = 3). **b** The top left pictures are representative fluorescence images in maximum relative fluorescence intensity of NC and HpeG2/NFAT2 cells and the top right bar graph is the statistical result of maximum fluorescence intensity in this two groups (*n* = 6). The bottom picture shows representative time-dependent relative fluorescence intensity changes of fluo-4-Ca^2+^ complex in NC and HpeG2/NFAT2 cells. Each bar represents the mean ± S.D.; ^*^p < 0.05, ^**^p < 0.01, ^***^p < 0.001 compared to the NC samples. Full-length blots are presented in Supplementary Figures

In order to detect the downstream signaling of NFAT2 transactivation, the co-stimulation of ionomycin and PMA was applied to stimulate HepG2 cells. The expression levels of Egr2 and FasL in HepG2/NFAT2 and NC cells were examined by three independent western blot experiments. The results indicated that, after the co-stimulation of ionomycin and PMA, the expression of Egr2 and FasL was increased, while the phosphorylation of AKT and ERK was decreased, in HepG2/NFAT2 cells compared with NC cells (Fig. 3a). The above results suggested that NFAT2 overexpression can facilitate the expression of anti-oncogene Egr2, apoptotic factor FasL and suppressed the activation of AKT and ERK.

### NFAT2 overexpression inhibited K^+^-induced Ca^2+^ mobilization

To investigate the anergic state of NFAT2 overexpressed, the calcium mobilisation assay was performed. As shown in Fig. 3b, the concentration of intracellular Ca^2+^ was immediately increased by 20 mmol/L K^+^ stimulation in HepG2 cells, which indicated that active Ca^2+^ influx occurred. Although the concentration of intracellular Ca^2+^ was also elevated in HepG2/NFAT2 cells, the increasement of Ca^2+^ amount was much less than that in NC cells. Furthermore, compared with NC cells, the fluorescence intensity (max) of HepG2/NFAT2 cells was remarkably low. The Live Cell Imaging analysis suggested that NFAT2 overexpression reduced Ca^2+^ mobilization in K^+^ stimulated HepG2 cells.

### The expression of NFAT2, Egr2, FasL, COX-2 and c-myc in tissue samples

The mRNA and protein expression of NFAT2, Egr2, FasL, COX-2 and c-myc was examined in 20 pairs of hepatic carcinoma tissues and adjacent nontumor tissues.

**Fig. 4:**
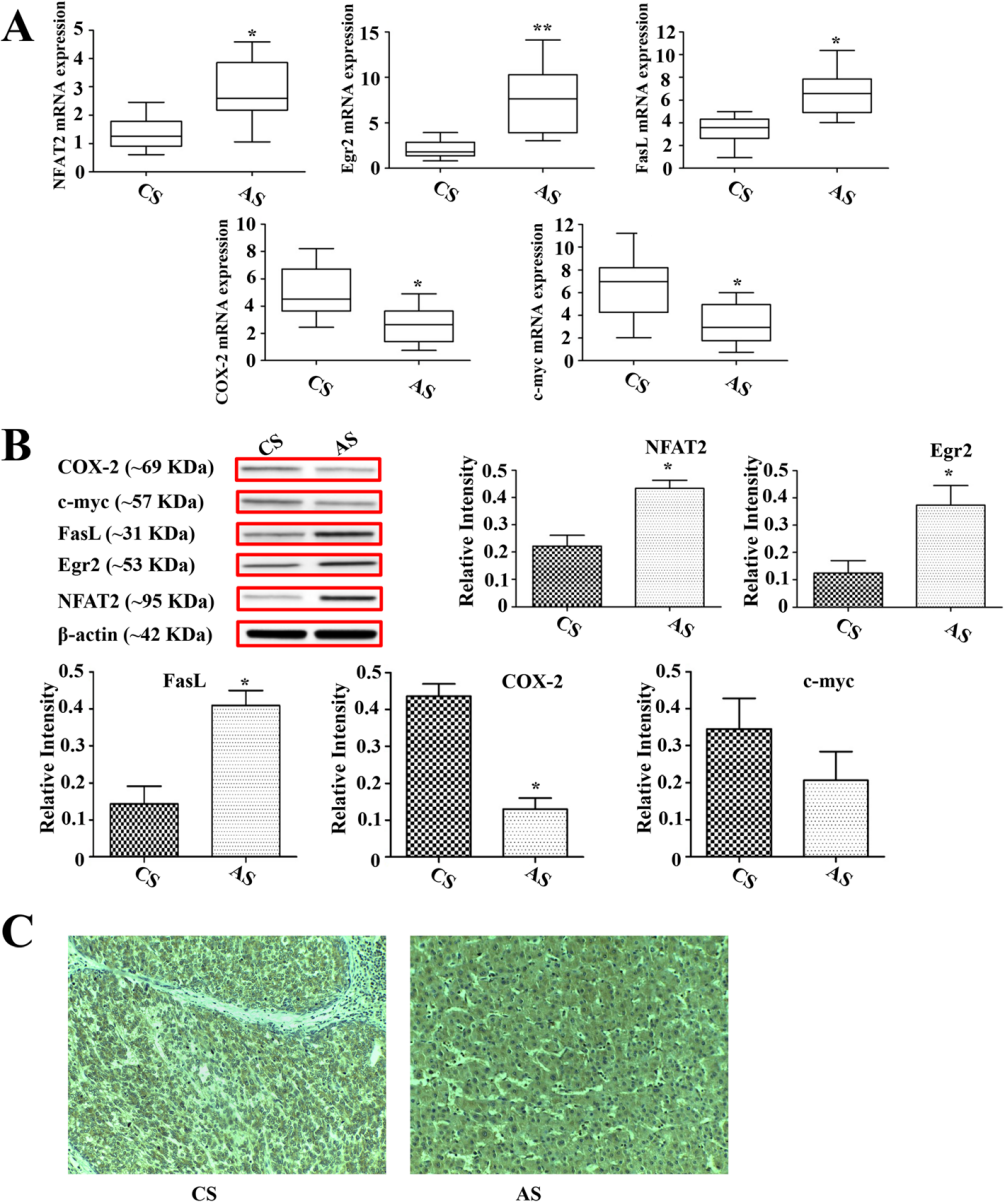
The mRNA and protein expression of NFAT2, Egr2, FasL, COX-2 and c-myc in hepatic carcinoma tissues and adjacent nontumor tissues. **a** The mRNA expression of NFAT2, Egr2, FasL, COX-2 and c-myc in hepatic carcinoma tissues and adjacent nontumor tissues (*n* = 20). **b** The protein expression of NFAT2, Egr2, FasL, COX-2 and c-myc in hepatic carcinoma tissues and adjacent nontumor tissues (*n* = 20). Each bar represents the mean ± S.D.; ^*^p < 0.05, ^**^p < 0.01, ^***^p < 0.001 compared to the carcinoma tissue samples. Full-length blots are presented in Supplementary Figures. **c** The representative images of immunohistochemical staining for NFAT2 in hepatic carcinoma tissue and adjacent nontumor tissue (Magnification, 200×).

Compared with adjacent nontumor tissues, the mRNA and protein expression of NFAT2, Egr2 and FasL in carcinoma tissues was obviously downregulated and the expression of COX-2 and c-myc was upregulated (Fig. 4a and b). Although the protein expression levels of c-myc in carcinoma tissues were higher than that in adjacent nontumor tissues, this upregulation showed no significant difference between two groups.

From the representative IHC images in Fig. 4c, hepatocytes in adjacent nontumor tissues extensively expressed NFAT2 in the cytoplasm and nuclei and those cells were stained as deep brown. Compared with hepatocytes in adjacent nontumor tissues, the cells in hepatic carcinoma tissues showed less immunostaining for NFAT2, which indicated week expression of NFAT2 in hepatic carcinoma tissues.

## Discussion

NFAT2 was originally regarded as a master modulator of T cell behaviors, such as proliferation, activation, exhaustion and anergy [19–21]. Some research proved that NFAT2 not only regulated T cell anergy, but exerted sensitivity accommodation to some tumor cells. In carboplatin resistant lung cancer cell line, NFAT2 knockdown and blocking its nucleus translation both could effectively restore the cell sensitivity to carboplatin [22]. Otherwise, in chronic myeloid leukaemia, NFAT2 high expression made a large contribution to develop cell resistance to tyrosine kinase inhibitors and NFAT2 ablation contributed to the transformation from indolent form to aggressive mode [23]. The sensitivity accommodation by NFAT2 in chronic myeloid leukaemia, has been proved to have close relationship with the inducing expression of anergy-associated genes such as Egr2, Grail and Lck [16]. In the current study, NFAT2 overexpression reduced the cell response to K^+^ stimulation in HepG2 cells, and the anergy-associated transcription factor Egr2 also highly expressed in HepG2/NFAT2 cells, which firstly suggested that NFAT2 overexpression inhibits the sensitive response of HepG2 cells to surrounding stimulations. Besides the sensitivity accommodation, upregulation of Egr2 also exerted inhibition effect on invasion and migration of gastric cancer [24], which was also observed in HepG2 cells in our research. Compared with adjacent nontumor tissues, the decreased expression of NFAT2 and Egr2 in carcinoma tissues was consistent with its aggression and malignancy.

FasL as a pivotal apoptosis factor triggers the extrinsic cell apoptosis [25] and some research indicated that NFAT2 could bind to the FasL promoter effectively inducing the expression of FasL, which was applied to explain the phenomenon that NFAT2 overexpression induced HCC cells apoptosis [18]. We did observe that NFAT2 overexpression in HepG2 cells inhibited cell viability and promoted cell apoptosis. Besides NFAT2, the expression of FasL was also modulated by the activated AKT and ERK. The activated AKT effectively inhibited the expression of FasL and promoted the cell survival through phosphorylating FOXO3 and then suppressing the transcriptions of its target genes [26]. Meanwhile, the fact that downregulated ERK signaling significantly promoted the expression of FasL and cell apoptosis in HCC, was also observed [27]. In our study, NFAT2 overexpression efficiently suppressed the activation of AKT and ERK, and thus, promoted the FasL expression. The NFAT2 expression in carcinoma tissues was downregulated while the FasL expression was also suppressed, which was in consistent with the fact observed in HepG2 cells.

COX-2 and c-myc always play harmful roles in the development of cancer. COX-2 plays vital role in facilitating tumor cells proliferation and angiogenesis in HCC [28], while c-myc modulates tumor cell cycle and promotes the growth of various tumor cells [29]. The absence of NFAT2 in carcinoma tissues would enhance the activation of AKT and ERK. The activated ERK could phosphorylate Ser62 site in c-myc, thus stabilizing the c-myc [30], while the activation of AKT promoted COX-2 expression and COX-2 dependent pro-metastasis [31]. In the current study, the expression of c-myc and COX-2 was obviously increased in carcinoma tissues, which was in agree with the previous research and our deduction.

## Conclusion

In conclusion, NFAT2 overexpression upregulated the expression of Egr2, FasL and suppressed the phosphorylation of AKT and ERK, which contributed to inducing cell apoptosis and inhibiting invasion and migration of HepG2 cells. Otherwise, we firstly demonstrated that NFAT2 overexpression suppressed Ca^2+^ mobilization in HepG2 cells stimulated by K^+^, through upregulating the expression of anergy-associated Egr2. The absence of NFAT2 and Egr2 in carcinoma tissues promotes sensitivity and malignancy of HCC. Therefore, our work suggests that NFAT2 is a promising therapeutic target for carcinoma sensitivity and aggression suppression in HCC treatment (Fig. S1).

## Supplementary information

**Additional file 1: Figure S1.** The potential mechanism of NFAT2 inducing anergic state. **Figure S2.** Full-length western blots.

## Data Availability

The datasets used and/or analysed during the current study are available from the corresponding author on reasonable request.

## Abbreviations

NFAT2: Nuclear factor of activated T cells 2
FasL: Fas ligand
COX-2: cyclooxygenase-2
c-myc: myc proto-oncogene protein
HCC: Hepatocellular carcinoma
CS: carcinoma samples
AS: adjacent nontumor samples
PI: propidine iodide
NC: negative control
ERK: extracellular-signal-related kinase
AKT: RAC serine/threonine-protein kinase
EdU: 5-ethynyl-2′-deoxyuridine

## References

[1] Ferlay J, Soerjomataram I, Dikshit R (2015). Cancer incidence and mortality worldwide: sources, methods and major patterns in GLOBOCAN 2012. Int J Cancer.

[2] Islami F, Miller KD, Siegel RL, Fedewa SA, Ward EM, Jemal A (2017). Disparities in liver cancer occurrence in the United States by race/ethnicity and state. CA Cancer J Clin.

[3] Craig AJ, Von Felden J, Villanueva A (2017). Molecular profiling of liver cancer heterogeneity. Discov Med.

[4] Shaw JP, Utz PJ, Durand DB, Toole JJ, Emmel EA, Crabtree GR (1988). Identification of a putative regulator of early T cell activation genes. Science.

[5] Tripathi P, Wang Y, Coussens M (2014). Activation of NFAT signaling establishes a tumorigenic microenvironment through cell autonomous and non-cell autonomous mechanisms. Oncogene.

[6] Flanagan WM, Corthesy B, Bram RJ, Crabtree GR (1991). Nuclear association of a T-cell transcription factor blocked by FK-506 and cyclosporine a. Nature.

[7] Buchholz M, Schatz A, Wagner M, Michl P, Linhart T, Adler G, Gress TM, Ellenrieder V (2006). Overexpression of c-myc in pancreatic cancer caused by ectopic activation of NFATc1 and the Ca^2+^/calcineurin signaling pathway. EMBO J.

[8] Viola JP, Carvalho LD, Fonseca BP, Teixeira LK (2005). NFAT transcription factors: from cell cycle to tumor development. Braz J Med Biol Res.

[9] Liu J, Jin P, Lin X, Zhou Q, Wang F, Liu S, Xi S (2018). Arsenite increase cyclin D1 expression through coordinate regulation of the Ca^2+^/NFAT2 and NF-κB pathways via ERK/MAPK in a human uroepithelial cell line. Metallomics.

[10] Lucena PI, Faget DV, Pachulec E, Robaina MC, Klumb CE, Robbs BK, Viola JP (2015). NFAT2 isoforms differentially regulate gene expression, cell death and transformation through alternative N-terminal domains. Mol Cell Biol.

[11] Liu J, Jin P, Lin X (2018). Arsenite increase cyclin D1 expression through coordinated regulation of the Ca^2+^/NFAT2 and NF-κB pathway via ERK/MAPK in a human uroepithelial cell line. Metallomics..

[12] Levin-Gromiko U, Koshelev V, Kushnir P (2014). Amplified lipid rafts of malignant cells constitute a target for inhibition of aberrantly active NFAT and melanoma tumor growth by the aminobisphosphonate zoledronic acid. Carcinogenesis..

[13] Quang CT, Leboucher S, Passaro D, Fuhrmann L, Nourieh M, Vincent-Salomon A, Ghysdael J (2015). The calcineurin/NFAT pathway is activated in diagnostic breast cancer cases and is essential to survival and metastasis of mammary cancer cells. Cell Death Dis.

[14] Tripathi MK, Deane NG, Zhu J (2014). Nuclear factory of activated T-cell activity is associated with metastatic capacity in colon cancer. Cancer Res.

[15] Xu W, Gu J, Ren Q, Shi Y, Xia Q, Wang J, Wang S, Wang Y, Wang J (2016). NFATC1 promotes cell growth and tumorigenesis in ovarian cancer up-regulating c-Myc through ERK1/2/p38 MAPK signal pathway. Tumour Biol.

[16] Märklin M, Heitmann JS, Fuchs AR (2017). NFAT2 is a critical regulator of the anergic phenotype in chronic lymphocytic leukaemia. Nat Commun.

[17] Xu S, Shu P, Zou S, Shen X, Qu Y, Zhang Y, Sun K, Zhang J (2018). NFATc1 is a tumor suppressor in hepatocellular carcinoma and induces tumor cell apoptosis by activating the FasL-mediated extrinsic signaling pathway. Cancer Med.

[18] Wang S, Kang X, Cao S, Cheng H, Wang D, Geng J (2012). Calcineurin/NFATc1 pathway contributes to cell proliferation in hepatocellular carcinoma. Dig Dis Sci.

[19] Rengarajan J, Tang B, Glimcher LH (2002). NFATc2 and NFATc3 regulate T(H) 2 differentiation and modulate TCR-responsiveness of naïve T(H) cells. Nat Immunol.

[20] Martinez GJ, Pereira RM, Äijő T (2015). The transcription factor NFAT promotes exhaustion of activated CD8(+) T cells. Immunity.

[21] Soto-Nieves N, Puga I, Abe BT, Bandyopadhyay S, Baine I, Rao A, Macian F (2009). Transcriptional complexes formed by NFAT dimers regulate the induction of T cell tolerance. J Exp Med.

[22] Liu X, Pan CG, Luo ZQ (2019). High expression of NFAT2 contributes to carboplatin resistance in lung cancer. Exp Mol Pathol.

[23] Gregory MA, Phang TL, Neviani P (2010). Wnt/Ca2+/NFAT signaling maintains survival of Ph+ leukemia cells upon inhibition of Bcr-Abl. Cancer Cell.

[24] Chen M, Fan L, Zhang SM (2019). LINC01939 inhibits the metastasis of gastric cancer by acting as a molecular sponge of miR-17-5p to regulate EGR2 expression. Cell Death Dis.

[25] Budihardjo I, Oliver H, Lutter M, Luo X, Wang X (1999). Biochemical pathways of caspase activation during apoptosis. Annu Rev Cell Dev Biol.

[26] Zhang XL, Zhuang TT, Liang ZY (2017). Breast cancer suppression by aplysin is associated with inhibition of PI3K/AKT/FOXO3a pathway. Oncotarget.

[27] Gong C, Fang J, Li G, Liu HH, Liu ZS (2017). Effects of microRNA-126 on cell proliferation, apoptosis and tumor angiogenesis via the down-regulating ERK signaling pathway by targeting EGFL7 in hepatocellular carcinoma. Oncotarget.

[28] Stasinopoulos I, O’Brien DR, Wildes F, Glunde K, Bhujwalla ZM (2007). Silencing of cyclooxygenase-2 inhibits metastasis and delays tumor onset of poorly differentiated metastatic breast cancer cells. Mol Cancer Res.

[29] Lee YS, Heo W, Son CH, Kang CD, Park YS, Bae J (2019). Upregulation of Myc promotes the evasion of NK cell-mediated immunity through suppression of NKG2D ligands in K562 cells. Mol Med Rep.

[30] Chen JR, Ding CF, Chen YH (2020). ACSL4 promotes hepatocellular carcinoma progression via c-Myc stability mediated by ERK/FBW7/c-Myc axis. Oncogenesis.

[31] Gan L, Qiu Z, Huang J (2016). Cyclooxygenase-2 in tumor-associated macrophages promotes metastatic potential of breast cancer cells through Akt pathway. Int J Biol Sci.

