# Supplementary material for: NFAT2 overexpression suppresses the malignancy of hepatocellular carcinoma through inducing Egr2 expression
Source: BMC Cancer. 2020 Oct 6;20:966. doi: 10.1186/s12885-020-07474-0 (PMC7542386; doi:10.1186/s12885-020-07474-0)

**NFAT2 overexpression suppresses the malignancy of hepatocellular carcinoma through inducing Egr2 expression**

Jian Wang^1^ MD, Yamin Zhang^1^* MD, Lei Liu^2^ MD, Zilin Cui^1^ MD, Rui Shi^1^ MD, Jianchun Hou^1^ MD, Zirong Liu^1^ MD, Long Yang^1^ MD, Lianjiang Wang^1^ MD, Yang Li^1^ MD

*^1^Hepatobiliary Surgery Department, Tianjin First Center Hospital, Tianjin Clinical Research Center for Organ Transplantation, Key Laboratory for Critical Care Medicine of the Ministry of Health, Tianjin, 300192, PR China.*

*^2^Department of Transplantation Center, Tianjin First Center Hospital, Tianjin Clinical Research Center for Organ Transplantation, Key Laboratory for Critical Care Medicine of the Ministry of Health, Tianjin, 300192, PR China.*

*Correspondence to: Yamin Zhang, Hepatobiliary Surgery Department, Tianjin First Center Hospital, Tianjin Clinical Research Center for Organ Transplantation, Key Laboratory for Critical Care Medicine of the Ministry of Health, No. 24 Fukang Road, Nankai District, Tianjin, 300192, PR China.

Email: [195156119@163.com](mailto:195156119@163.com)

ORCID (Jian Wang): 0000-0002-8411-8983

Running Head: NFAT2 suppresses the malignancy of hepatoma

Figure S1. The potential mechanism of NFAT2 inducing anergic state.


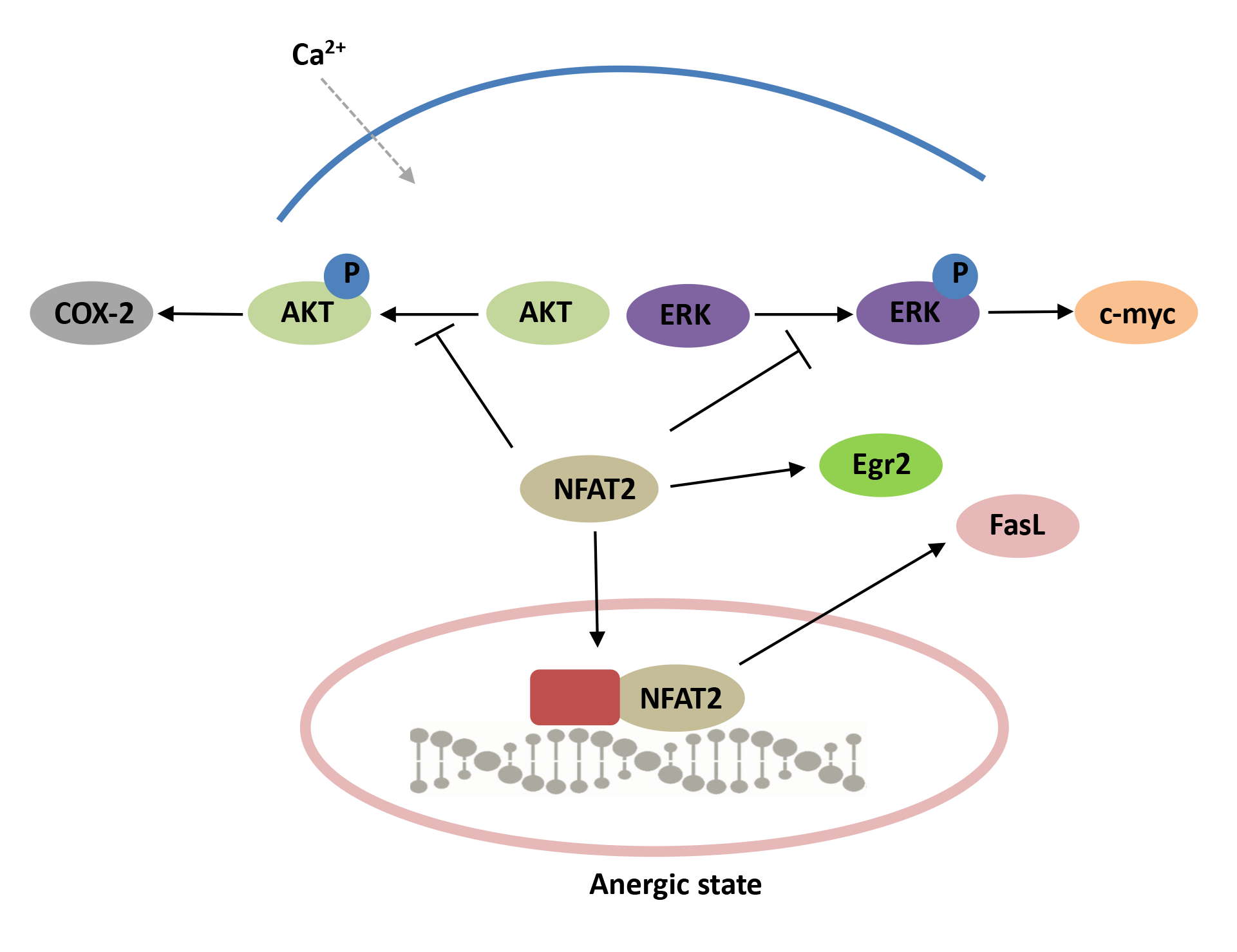


Figure S2. Full-length western blots


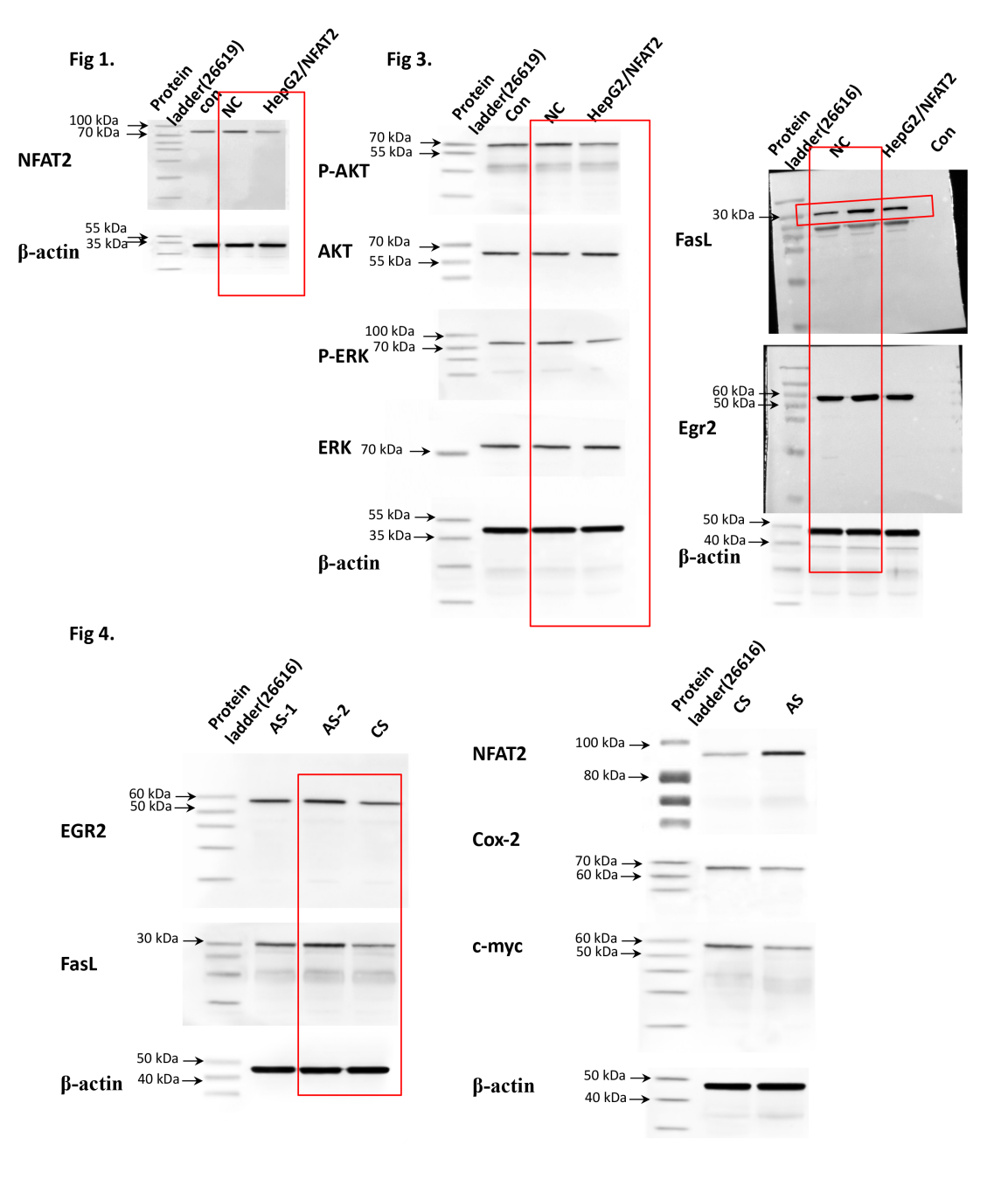

Supplement: Supplementary file 1 — Additional file 1: Figure S1. The potential mechanism of NFAT2 inducing anergic state. Figure S2. Full-length western blots. [file 12885_2020_7474_MOESM1_ESM.docx]
